# Mechanisms of tauroursodeoxycholate-mediated inhibition of apoptosis

**DOI:** 10.1186/2047-783X-19-S1-S22

**Published:** 2014-06-19

**Authors:** Annika Sommerfeld, Patrick GK Mayer, Roland Reinehr, Dieter Häussinger

**Affiliations:** 1 Clinic of Gastroenterology, Hepatology and Infectious Diseases, Heinrich Heine University, 40225 Düsseldorf, Germany

## 

The hydrophilic bile acid ursodeoxycholic acid (UDCA), which *in vivo* is converted to its taurine conjugate tauroursodeoxycholic acid (TUDC), is a mainstay in the treatment of cholestatic liver disease. TUDC prevents bile acid-induced cholestasis and induces choleresis. Its beneficial effects involve an increased hepatobiliary secretion as well as cytoprotective and antiapoptotic effects. The choleretic effect of TUDC largely depends on a rapid targeting and insertion of intracellularly stored transport ATPases into the canalicular membrane, such as the bile salt export pump (Bsep) and multidrug resistance protein-2 (Mrp2) [1]. It was recently shown that the TUDC-induced signaling involves phosphorylation of the epidermal growth factor receptor (EGFR) at tyrosine 845 and 1173, but not Tyr1045. Tyr845, a known Src kinase target, triggers an activating autophosphorylation at Tyr1173 of the EGFR [2]. Furthermore, c-Src, focal adhesion kinase (FAK), phosphatidylinositol 3-kinase (PI3K) and Ras/Raf are activated within minutes by TUDC, which triggers downstream dual activation of extracellular signal-regulated kinases (Erks) and p38 mitogen-activated protein kinase (p38^MAPK^) [1-4].

Recent data indicate that integrins are a long-searched sensor for TUDC in the liver, because TUDC interacts with α_5_ß_1_ integrins in the liver resulting in a conformational change with activation of ß_1_ integrin and the initiation of downstream integrin signaling. In contrast to the previously reported ß_1_ integrin activation in response to hypoosmolarity or insulin, TUDC-induced integrin activation occurs inside the hepatocyte and requires TUDC uptake via the basolateral Na^+^/taurocholate cotransporting polypeptide (Ntcp) [2]. However, other mechanisms of TUDC action may contribute to its choleretic effect under pathological cholestatic conditions.

Hepatocyte apoptosis can be triggered by the retention of hydrophobic bile acids in hepatocytes by activation of ligand-independent death receptor pathways *in vivo* and *in vitro*. Toxic bile acids such as taurolithocholyl-3 sulfate (TLCS) and glycochenodeoxycholate (GCDC) have previously been shown to induce apoptosis in a CD95-dependent apoptosis, involving activation of the acidic sphingomyelinase, increased ceramide formation and activation of the protein kinase Cζ (PKCζ) followed by an oxidative stress response via serine phosphorylation of p47^phox^ and NADPH oxidase activation. Putative inhibition of phosphatase activities by reactive oxygen species (ROS) triggers an activation of the Src family kinase Yes and activation of the c-Jun-N-terminal kinase-1/-2 (JNK). The activated Yes kinase associates with the EGFR resulting in a Yes-mediated EGFR transactivation. Activated EGFR then associates in a JNK-dependent way with CD95 and catalyzes CD95 tyrosine phosphorylation, which triggers CD95 membrane trafficking, death-inducing signaling complex (DISC) formation, i.e. association of Fas-associated death domain (FADD) and caspase 8 to the death receptor, followed by caspase 8-dependent mitochondrial amplification of pro-apoptotic signaling, which finally leads to apoptotic cell death [5].

Bile salt-induced apoptosis can be antagonized by TUDC. This cytoprotective effect of TUDC results from an inhibition of JNK activation and CD95 trafficking to the cell surface and activation of caspases 8 and 9 [6]. However, the molecular basis of TUDC-sensing with regard to anti-apoptosis is not yet settled, but may involve a TUDC-mediated inhibition of bile salt-induced phosphorylation of the MAP kinase kinase 4/7 (MKK4/7) upstream of JNK activation Furthermore, similar to cAMP, TUDC induced a protein kinase A (PKA)-sensitive serine/threonine phosphorylation of the CD95 in presence of GCDC, which was identified as an internalization signal for CD95. Inhibition of hepatocyte apoptosis by cAMP involves both PKA-dependent serine/threonine phosphorylation of the CD95 and inhibition of EGFR activation, which results in an inhibition of CD95 tyrosine phosphorylation, CD95 membrane targeting, and DISC formation. mRNA analyses show that the dual specificity MAP kinase phosphatase MKP-1, a transcriptionally regulated immediate early gene-encoded enzyme, is upregulated in response to TUDC after stimulation with GCDC for up to 60 min, which was already shown for cAMP [7]. Preliminary data suggest that TUDC-induced integrin activation is involved in mediating its anti-apoptotic effect.
